# Telomere-Associated Proliferative Capacity in Expandable Porcine Hepatocyte-like Progenitor Cells

**DOI:** 10.3390/biology15120958

**Published:** 2026-06-18

**Authors:** Sun A Ock, Yeongji Kim, Imran Ullah, Young-Im Kim, Ran Lee, Keon Bong Oh, Seongsoo Hwang, Juyoung Lee

**Affiliations:** 1 Animal Biotechnology and Genomics Division, National Institute of Animal Science, Rural Development Administration, 1500 Kongjwipatjwi-ro, Isero-myeon, Wanju-gun 565-851, Jeonbuk-do, Republic of Koreakeonoh@korea.kr (K.B.O.); hwangss@korea.kr (S.H.);; 2 Department of Biochemistry, Faculty of Biological Sciences, Quaid-i-Azam University, Islamabad 15320, Pakistan

**Keywords:** hepatocyte-like progenitor cells, direct reprogramming, porcine fibroblasts, proliferation, telomere

## Abstract

Primary hepatocytes are limited by poor proliferative capacity and a finite replicative lifespan, restricting their utility in long-term in vitro studies. Here, we report the generation of expandable hepatocyte-like progenitor cells from GGTA1 knockout pigs, a large-animal model with reduced immunogenicity. Porcine fibroblasts were directly reprogrammed using a non-integrative episomal system encoding hepatic transcription factors, enabling stable lineage conversion without genomic integration. A simplified two-vector configuration combined with codon optimization enabled evaluation of vector-dependent effects while maintaining genomic safety without viral integration. The resulting cells exhibited hepatocyte-like morphology and gene expression, and transcriptomic analysis revealed a progressive shift toward liver-associated profiles during extended culture. Chromosomal analysis revealed vector-dependent differences in genomic stability, with codon-optimized cells showing increased aneuploidy, indicating a trade-off between proliferative capacity and genomic integrity. The cells also demonstrated sustained proliferative capacity, supported by maintenance of telomere length, increased expression of *TERT* and *MYC*, and reduced *CDKN1A* levels. Importantly, sustained proliferation was supported by complementary evidence from chromosomal and telomeric analyses. Although chromosomal alterations were observed during long-term culture, their biological significance remains to be fully determined. These cells partially recapitulate hepatocyte functions and provide a renewable in vitro system for studies of hepatic biology, proliferation, drug metabolism, toxicity, and repeated in vitro applications.

## Simple Summary

Liver cells are widely used in research to study metabolism and drug responses, but primary hepatocytes have limited ability to grow and survive over long periods. To overcome this limitation, we developed a new type of expandable liver-like cell using pig cells. Pigs are considered a valuable model because their biology is closer to humans than that of rodents. In this study, we converted pig fibroblasts into hepatocyte-like progenitor cells using a non-integrating gene delivery method, which avoids permanent genetic modification. The resulting cells showed key features of liver cells and were able to grow continuously over time. Importantly, their growth capacity was supported by stable chromosomal characteristics, indicating maintained genomic stability during long-term culture. This was further supported by maintenance of telomere length, which is essential for sustained cell proliferation. Although these cells do not fully match mature liver cells, they provide a stable and renewable system that can be used repeatedly in laboratory studies. This model may be useful for studying liver biology, testing drug safety, and reducing the need for animal experiments.

## 1. Introduction

The liver, as the central metabolic organ of the body, has a remarkable regenerative capacity in vivo but fails to maintain function long-term under ex vivo conditions [1,2,3]. Despite advances in clinical management, maintaining hepatocyte function and proliferation in vitro remains a major challenge, highlighting the need for stable and expandable hepatic cell systems [4,5].

Hepatocyte transplantation and related approaches have been explored; however, their application is limited by the scarcity of viable cells and rapid functional decline after isolation [1,5]. Under standard two-dimensional (2D) monolayer culture conditions, mature hepatocytes show a marked decline in proliferative capacity, quickly lose hepatic function, are prone to spontaneous cell death, and respond poorly to cryopreservation [2]. These limitations make it difficult to establish a reliable and expandable hepatic cell source for long-term studies.

Efforts to overcome these limitations have included the generation of hepatocyte-like cells from embryonic stem cells (ESCs) or induced pluripotent stem cells (iPSCs) [6,7]. While these cells resemble primary hepatocytes in many aspects, differentiation protocols remain costly, labor-intensive, and time-consuming. In addition, ESC-based methods raise ethical concerns. Direct conversion of somatic cells using liver-enriched transcription factors provides a more efficient and straightforward alternative that bypasses pluripotency [8,9,10,11]. However, a key limitation of current hepatic models is the inability to maintain long-term proliferative capacity while preserving hepatic characteristics. Therefore, establishing expandable hepatic cell systems remains a critical objective for in vitro liver research.

In this study, we used somatic cells derived from alpha1,3-galactosyltransferase knockout pigs, an established animal model in xenotransplantation, to generate expandable hepatocyte-like progenitor cells. The absence of the α-Gal epitope enhances immunological compatibility by reducing innate antibody-mediated recognition in humans, thereby providing a relevant large-animal source for hepatic cell studies [12,13]. Using a non-integrative episomal vector system with porcine-optimized hepatic transcription factors, we established a reprogramming strategy that enables sustained cellular expansion. The resulting cells exhibited long-term proliferative capacity accompanied by telomere maintenance and modulation, supporting enhanced cellular expansion. Together, this study presents an expandable hepatic cell platform derived from a large-animal model, providing a useful system for investigating hepatic cell biology and long-term proliferative dynamics in vitro.

## 2. Materials and Methods

### 2.1. Reagents and Media

Unless otherwise specified, all chemicals were purchased from Sigma-Aldrich Corporation (St. Louis, MO, USA), and media were obtained from Thermo Fisher Scientific (Waltham, MA, USA).

### 2.2. Experimental Animals and Fibroblast Isolation

All experiments were performed under the guidelines of the Institutional Animal Care and Use Committee of the National Institute of Animal Science (NIAS), Rural Development Administration, Republic of Korea (IACUC approval no. NIAS-2015-0155; Approval date: 16 September 2015). The experimental animal was a 1-month-old male GGTA1 (α1,3-galactosyltransferase)-knockout (GalTKO) pig developed and maintained at the NIAS. Fibroblasts derived from the ear tissue of this pig were cultured in vitro with Advanced DMEM supplemented with 10% FBS and used for the experiments. Additionally, fibroblasts from a conventional wild-type pig were included for comparison.

### 2.3. Episomal Vector Construction and Delivery

For episomal reprogramming, codon-optimized episomal vectors were engineered to enhance the expression of hHNF1A, hHNF4A, and hFOXA3 in porcine cells by condensing the transcription factors into two constructs (hHNF1A and hHNF4A-F2A-hFOXA3) (Invitrogen, Thermo Fisher Scientific) (Supplementary Table S1). Non-codon-optimized control vectors were synthesized in parallel. Episomal vectors were delivered using Lipofectamine 3000 according to the manufacturer’s instructions (Invitrogen, Thermo Fisher Scientific).

For comparison, lentiviral vectors encoding the same transcription factors were applied to wild-type porcine fibroblasts (non-GalTKO) at a multiplicity of infection (MOI) of 2 for 24 h, serving as an auxiliary control to confirm that the observed cellular changes were not specific to the episomal system or genetic background [11,14].

### 2.4. Induction of Porcine Induced Hepatocyte-like Cells (piHeps)

Fibroblasts derived from GalTKO pigs were used as the primary cell source, with wild-type fibroblasts serving as controls. For reprogramming, cells were pretreated with 1 μM 5-azacytidine for 24 h to enhance epigenetic accessibility. Episomal vectors (stock concentration: 1 mg/mL) were delivered using Lipofectamine 3000 in antibiotic-free advanced DMEM supplemented with 2% FBS for 24 h, following the manufacturer’s transfection protocol. After vector delivery, cells were cultured under hepatic induction conditions and monitored for lineage conversion by assessing morphological changes and hepatic gene expression. The induction medium was prepared as previously described and refreshed every three days [11]. To examine whether inhibition of TGF-β signaling enhances hepatic induction efficiency, A-83-01 (2 μM) was added to the culture medium.

### 2.5. Gene Expression Analysis and Genotype Validation

Quantitative real-time PCR was performed to assess gene expression levels. Total RNA was extracted, and cDNA synthesis and qPCR reactions were carried out following previously described protocols [11]. *HPRT1* was used as the internal reference gene, and all samples were analyzed in quintuplicate. Detailed primer sequences and the corresponding accession numbers are provided in Supplementary Table S2.

Genotype validation of GalTKO pigs and their derived piHeps was performed by genomic PCR and agarose gel electrophoresis as previously described [12], and genotype-specific band patterns were confirmed accordingly.

### 2.6. Immunocytochemical Fluorescence Staining for Hepatocyte-Specific Proteins

Cell pretreatment and immunostaining procedures were performed as described in our previous study [11], and detailed information on the primary and secondary antibodies used is provided in Supplementary Table S3. Stained samples were imaged using either a confocal or fluorescence microscope (Leica DMI6000B, Wetzlar, Germany). To assess α-Gal epitope expression in piHeps, cells derived from both GalTKO pigs and wild-type pigs were subjected to immunofluorescence staining as previously described [12]. Hepatocyte-like protein expression was assessed by co-staining for albumin, and nuclei were counterstained with DAPI.

### 2.7. In Vitro Characterization of Hepatocyte-like Features

Histochemical and cellular assays were performed to evaluate hepatocyte-like characteristics. Neutral lipid accumulation was assessed by Oil Red O staining, and glycogen storage was examined using periodic acid–Schiff (PAS) staining (Millipore, Burlington, MA, USA). Cellular uptake and release of indocyanine green (ICG) and acetylated low-density lipoprotein (ac-LDL) were also examined. All procedures were conducted according to previously reported protocols and the manufacturer’s instructions [11,15].

### 2.8. Urea Secretion Assay

To assess hepatocyte-like characteristics, urea levels in the culture supernatant were measured. Cell culture media from piHeps were collected on days 2 and 3 after seeding (day 0), and hepatocyte induction medium (HIM) was used as a control. Urea concentrations were determined using a Urea Assay Kit (Abcam, Cambridge, UK), and absorbance was measured with an iMark™ Microplate Absorbance Reader (Bio-Rad, Hercules, CA, USA) using Microplate Manager 6 software. Each experiment was performed in triplicate.

### 2.9. Genome-Wide Gene Expression Profiling Using Microarray

Total RNA (1 μg) was extracted from ear fibroblasts (negative control), piHeps matured for 1 and 3 months, and adult pig liver (positive control). Both wild and CO piHeps were subjected to the same maturation induction protocol, although passage numbers varied due to vector-dependent growth differences. Genome-wide gene expression profiling was performed by Macrogen Inc. (Seoul, Republic of Korea) using microarray analysis. Expression data were normalized to EF levels. Independent reprogramming batches refer to experiments initiated on different days using fibroblasts isolated from the same donor pig.

### 2.10. Karyotyping and Ploidy Analysis of piHeps

Karyotype analysis was performed on 30 piHep cells per group at passages 12 and beyond using the GTG-banding method, conducted by a specialized chromosome analysis service (Korea Research of Animal Chromosomes, Seoul, Republic of Korea). Cell-cycle ploidy assessment was performed as previously described [11], and the proportions of diploid and polyploid populations were determined accordingly.

### 2.11. Telomere Length Measurement

Telomere length was quantified using the Absolute Pig Telomere Length Quantification qPCR Assay Kit (ScienCell Research Laboratories, Carlsbad, CA, USA). A reference pig genomic DNA sample (Lot #26516; telomere length: 371 ± 29 kb per diploid cell) was included for assay calibration. Genomic DNA samples were analyzed using 2 ng of input DNA per reaction, based on optimization tests confirming accurate measurement within this range.

### 2.12. Statistical Analysis

All assays were performed in technical triplicate unless otherwise indicated. Statistical analyses were conducted using IBM SPSS Statistics (version 24; IBM Corp., Armonk, NY, USA). For comparisons among multiple groups, one-way analysis of variance (ANOVA) followed by Tukey’s post hoc test was applied. Comparisons between two groups were performed using an independent *t*-test. Quantitative PCR (qPCR) data are reported as relative quantification (RQ) values with corresponding minimum and maximum ranges, whereas all other data are presented as mean ± standard error of the mean (SEM). A *p*-value < 0.05 was considered statistically significant.

## 3. Results

### 3.1. Derivation and Characterization of Porcine Induced Hepatocyte-like Cells

**Figure 1:**
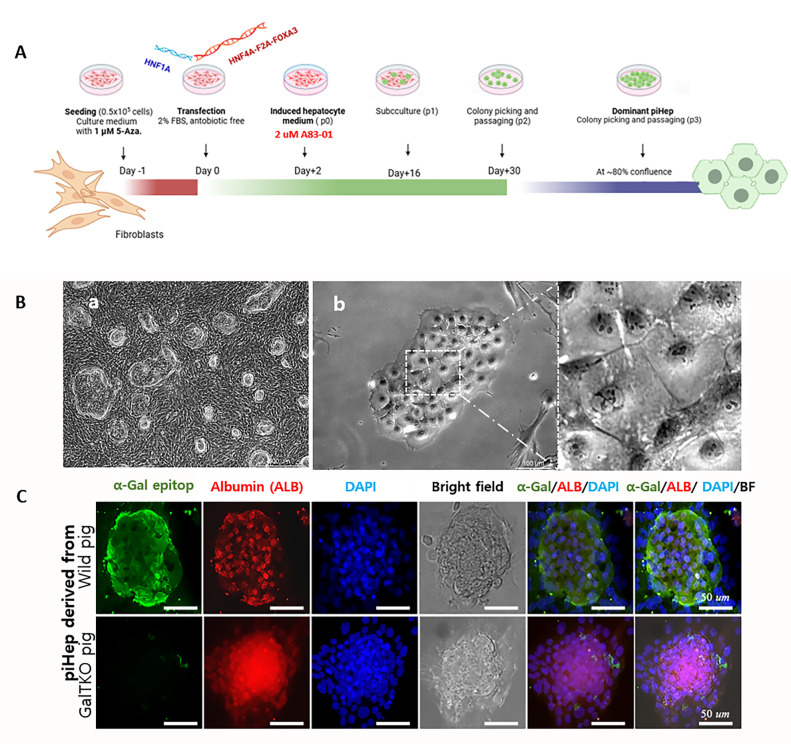
Generation and characterization of porcine induced hepatocyte-like cells (piHeps) from GalTKO fibroblasts. (**A**) Schematic overview of the piHep induction protocol. Porcine ear fibroblasts were pretreated with 1 μM 5-azacytidine for 24 h (Day −1), followed by vector delivery on Day 0. Cells were subsequently cultured under hepatic induction conditions, subcultured on Day 16 (p1), and single-cell-derived colonies were established at P2 and expanded at p3. (**B**) Morphological changes during reprogramming. (**a**) Representative epithelial-like colonies at P2. Scale bar, 500 μm. (**b**) piHeps at p3 exhibiting polygonal morphology with enlarged nuclei; binucleated cells are indicated (dashed box). Scale bar, 100 μm. (**C**) Confocal immunofluorescence analysis of α-Gal epitope (green) and albumin (red) in piHeps derived from wild and GalTKO pigs. α-Gal and albumin were detected in wild piHeps, whereas only albumin was observed in GalTKO piHeps. Nuclei were counterstained with DAPI (blue). Scale bar, 50 μm.

Porcine induced hepatocyte-like cells (piHeps) were generated by introducing two episomal vectors encoding human HNF1A and HNF4A-F2A-FOXA3 into ear fibroblasts derived from GalTKO pigs. To evaluate the effect of codon optimization, both codon-optimized and non-optimized episomal vectors were introduced into fibroblasts derived from GalTKO and wild pigs (Figure 1A). Two weeks after transfection, cells were subcultured, and emerging colonies (Figure 1B(a)) were isolated and expanded from single cells. The resulting cells exhibited epithelial-like morphology, including a polygonal shape, abundant cytoplasm, large round nuclei, and occasional binucleation (Figure 1B(b)).

Immunofluorescence staining demonstrated the expression of hepatic-associated markers, including albumin, alpha-1-antitrypsin, transferrin, and E-cadherin in piHeps. Drug-metabolizing enzymes, including CYP1A1, CYP2A, and CYP3A1, showed partial positive signals, whereas CYP3A29 exhibited relatively higher expression (Supplementary Figure S1). Based on these morphological and phenotypic characteristics, we confirmed the successful induction of piHeps. Albumin expression was consistently detected in all piHeps, whereas the α-Gal epitope was markedly absent in GalTKO-derived piHeps compared with wild piHeps (Figure 1C). Genotypic analysis further confirmed the targeted gene disruption in GalTKO-derived piHeps (Supplementary Figure S2), demonstrating that the induced cells faithfully retained the genetic identity of their donor fibroblasts.

### 3.2. Vector-Associated Changes in Hepatic Gene Expression

**Figure 2:**
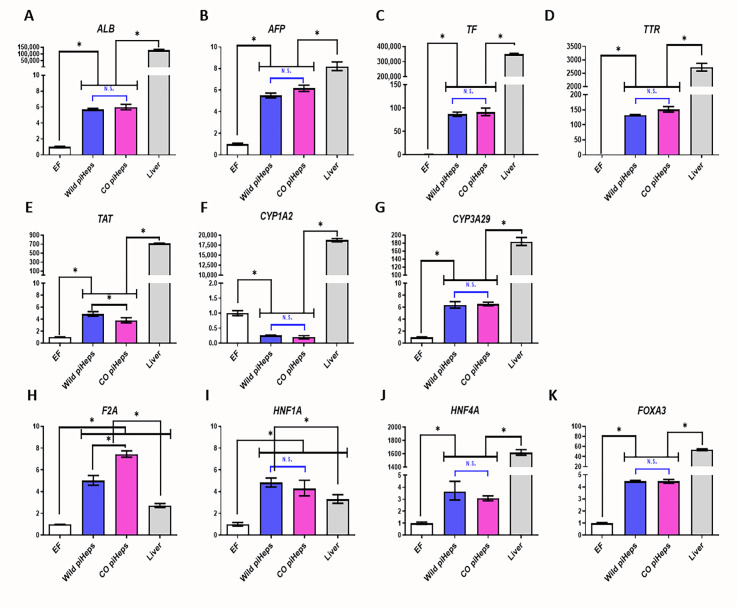
Expression of hepatic-associated and transcriptional genes in piHeps. Gene expression analysis was performed four weeks after reprogramming. Ear fibroblasts (EF) served as negative controls, and porcine liver tissue served as positive controls (gray bars). Blue bars indicate wild piHeps generated using non-codon-optimized vectors, and violet bars indicate CO piHeps generated using codon-optimized vectors. (**A**–**E**) Hepatic-associated genes (*ALB*, *AFP*, *TF*, *TTR*, *TAT*). (**F**,**G**) Xenobiotic-metabolizing enzymes (*CYP1A2*, *CYP3A29*). (**H**) *F2A* expression indicating residual vector presence. (**I**–**K**) Endogenous porcine hepatic transcription factors (*HNF1A*, *HNF4A*, *FOXA3*). Data are presented as relative quantification (RQ) values with minimum and maximum ranges. Experiments were performed in technical triplicate to ensure reproducibility. Statistical significance was determined by one-way ANOVA followed by Tukey’s post hoc test, and brackets indicate the specific groups compared. * *p* < 0.05; N.S., not significant.

After four weeks of differentiation induction, hepatic gene expression profiles were analyzed in vector-introduced piHeps (Figure 2). mRNA expression of albumin (*ALB*) and alpha-fetoprotein (*AFP*) was increased in piHeps irrespective of codon optimization. Although *ALB* expression remained lower than that in liver tissue, transferrin (*TF*) and transthyretin (*TTR*) were significantly upregulated compared with ear fibroblast (EF) controls. Tyrosine aminotransferase (*TAT*) expression was also increased, with wild piHeps showing slightly higher levels than CO piHeps (Figure 2A–E).

Expression of drug-metabolizing enzymes was further examined. *CYP1A2* showed minimal changes, whereas *CYP3A29* expression was increased relative to EF controls regardless of codon optimization (Figure 2F,G). Expression of F2A, indicative of exogenous vector presence, was detected in both groups but was significantly reduced after three months of culture (Figure 2H; Supplementary Figure S3A). Notably, *F2A* expression levels were higher in CO piHeps than in wild piHeps. In parallel, endogenous porcine *HNF1A* expression was increased, and *HNF4A* and F*OXA3* were also upregulated (Figure 2I–K).

To assess hepatic-like features, histochemical assays were performed. Glycogen accumulation (PAS staining) and ICG uptake and clearance were observed in piHeps, indicating the acquisition of limited hepatic-associated characteristics (Supplementary Figure S3). Urea secretion was also increased in piHeps compared with EF controls, with higher levels observed in CO piHeps than in wild piHeps.

### 3.3. Transcriptomic Changes in piHeps During Culture Progression

**Figure 3:**
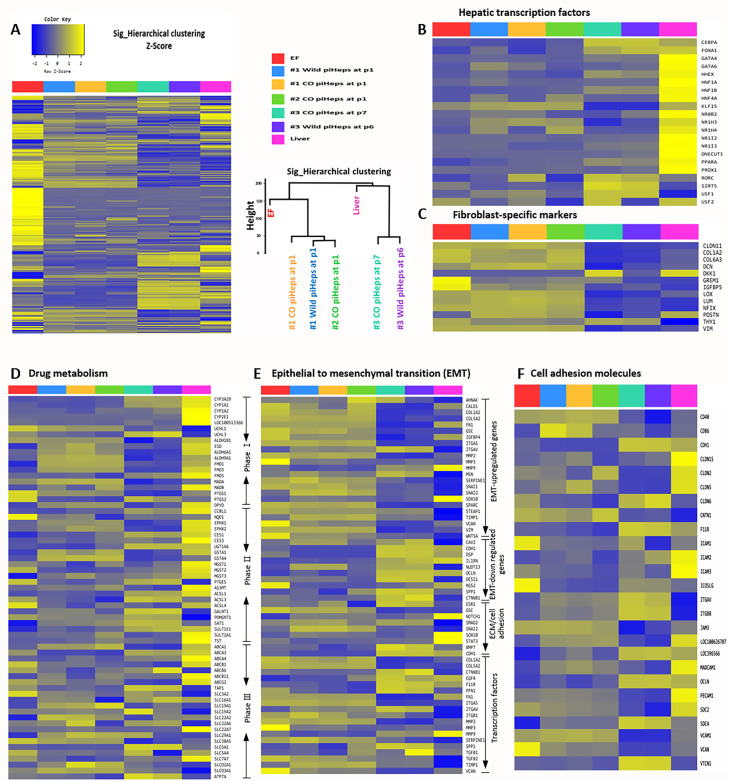
Transcriptomic profiling of hepatic-like gene expression changes in piHeps. piHeps were generated from ear fibroblasts (EF) using either wild or codon-optimized (CO) episomal vectors and analyzed at early (4 weeks) and late (3 months) stages of differentiation. (**A**) Microarray-based global gene expression analysis displayed as a heatmap (**left**) and hierarchical clustering (**right**), illustrating time-dependent transcriptomic shifts toward liver-associated profiles. (**B**–**F**) Heatmaps showing the relative expression of gene sets associated with (**B**) hepatic transcription factors; (**C**) fibroblast-specific markers; (**D**) Phase I, II, and III drug metabolism-related genes; (**E**) epithelial–mesenchymal transition (EMT); and (**F**) cell adhesion molecules. A two-fold change cutoff was applied to define differential expression. Sample groups include EF (negative control), porcine liver (positive control), and piHeps from independent batches at early (p1) and late (p6–p7) passages. In the heatmaps, the color scale represents the relative Z-score of gene expression, where yellow indicates upregulation and blue indicates downregulation.

Microarray analysis performed at one and three months post-differentiation revealed time-dependent changes in global gene expression profiles in both wild and CO piHeps (Figure 3A). Heatmap visualization and hierarchical clustering indicated that gene expression patterns in piHeps progressively shifted toward liver-associated profiles over time.

At one-month post-differentiation, hepatic-associated transcription factors, including HNF4A, NR1H3, and GATA6, were expressed at levels distinct from those in fibroblasts. By three months, additional regulators such as CEBPA, FOXA1, HHEX, and PPARA showed increased expression (Figure 3B). Genes associated with the fibroblast state were reduced over time, whereas epithelial-associated markers, including CDH1 and OCLN, were upregulated, consistent with a transition toward an epithelial-like phenotype (Figure 3E,F).

Expression of representative metabolic and transport-related genes also showed increasing trends during culture progression (Figure 3D; Supplementary Figure S4). In addition, quantitative PCR analysis confirmed increased expression of CYP3A29 and CYP1A2, with relatively higher levels observed in CO piHeps (Supplementary Figure S5).

Overall, these results indicate a progressive shift in gene expression from fibroblast-associated profiles toward hepatic-like characteristics during extended culture.

### 3.4. Vector-Associated Changes in Chromosomal Stability and Cell-Cycle Dynamics

**Figure 4:**
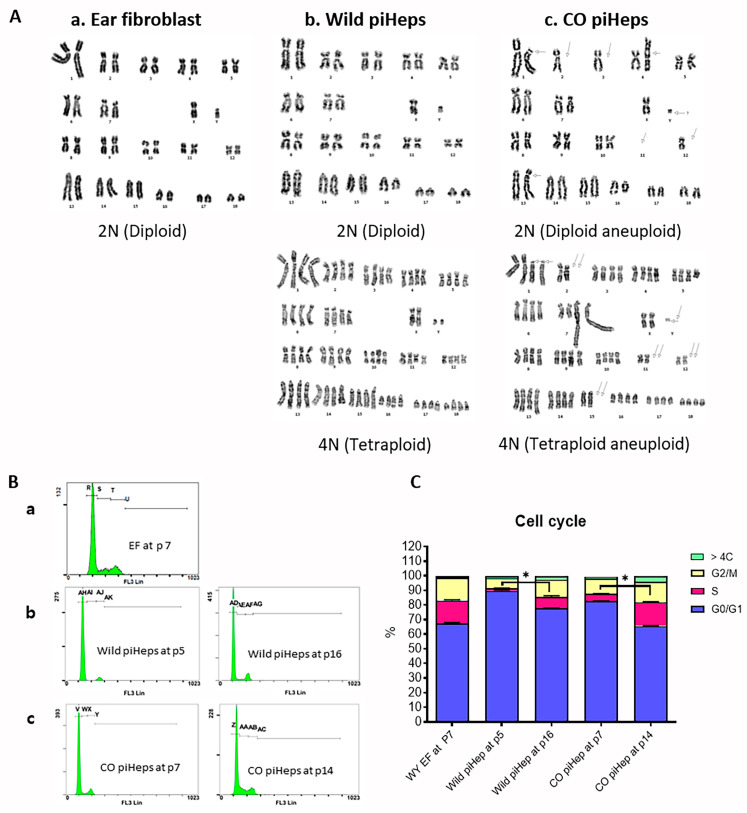
Vector-associated chromosomal stability and cell-cycle characteristics of piHeps. (**A**) Representative karyotype analysis of (**a**) EF, (**b**) wild piHeps, and (**c**) CO piHeps assessed by GTG banding. Arrows indicate chromosomal abnormalities, including chromosomal loss, partial deletions of the long (q) arm, or insertion of chromosomal fragments, reflecting aneuploidy in certain populations. (**B**) Representative DNA content histograms of (**a**) EF at p7, (**b**) wild piHeps at p5 (**left**) and p16 (**right**), and (**c**) CO piHeps at p7 (**left**) and p14 (**right**), illustrating changes in proliferative and ploidy profiles during long-term culture. (**C**) Cell-cycle analysis showing the distribution of cells across the G0/G1 (blue), S (red), G2/M (yellow), and >4C (green, representing polyploid cells) phases. Data in (**C**) are presented as the mean from three independent culture replicates. Asterisks (*) indicate statistically significant differences specifically in the S phase between the compared groups (*p* < 0.05).

Karyotype analysis was performed on piHeps at passage 10 or higher, together with chromosomal analysis of the original cells prior to vector introduction (Figure 4). Wild piHeps, generated using non-codon-optimized vectors, predominantly maintained a diploid karyotype, with only a minor population of tetraploid cells detected. In contrast, CO piHeps exhibited extensive aneuploidy within diploid cell populations, and tetraploid cells also displayed aneuploidy, although at a lower frequency.

Cell-cycle analysis across serial passages further revealed sustained proliferative activity in piHeps compared with EFs (Figure 4B,C). In Wild piHeps, the proportion of S-phase cells increased 4.5-fold with extended culture, rising from 1.72 ± 0.24% at p5 to 7.81 ± 0.95% by p16 (*p* < 0.05). CO piHeps exhibited a more rapid and robust expansion, with the S-phase population increasing 3.3-fold from 4.95 ± 0.29% at p7 to 16.40 ± 0.29% by p14 (*p* < 0.05), reaching a level comparable to EFs at p7 (15.50 ± 0.54%). At later passages, piHeps exhibited an increase in the proportion of cells with polyploid DNA content (>4C). Specifically, this population rose from 1.59% to 2.61% in Wild piHeps and from 1.16% to 3.61% in CO piHeps (*p* < 0.05). Notably, despite this upward trend, the absolute percentage of >4C cells remained remarkably low (less than 4%) throughout the long-term culture period, indicating that piHeps maintain relatively stable ploidy dynamics during extensive expansion (Figure 4C).

### 3.5. Telomere-Associated Proliferative Changes During Long-Term Culture

**Figure 5:**
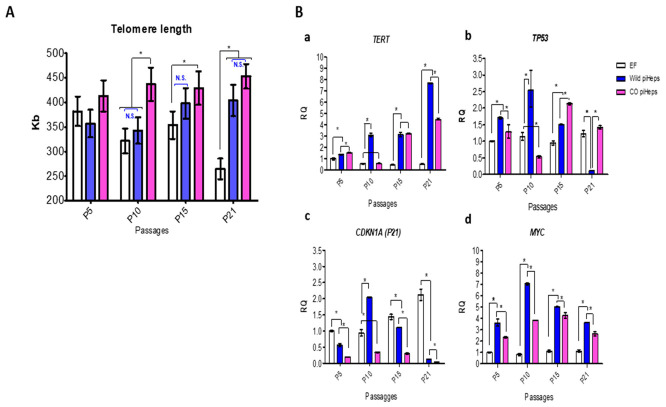
Telomere-associated extension of proliferative capacity in long-term cultured piHeps. piHeps were serially cultured in vitro up to passage 21. Telomere length and expression of proliferation-associated genes were analyzed at passages 5, 10, 15, and 21, with experiments performed in triplicate. (**A**) Telomere length measurements across serial passages. The *y*-axis indicates telomere length (kilobases, kb), and the *x*-axis indicates passage number. (**B**) Relative expression of proliferation-related genes, including (**a**) *TERT*, (**b**) *TP53*, (**c**) *CDKN1A (p21)*, and (**d**) *MYC*. Data are presented as relative quantification (RQ) values with minimum and maximum ranges. Open bars indicate ear fibroblasts (EF), blue bars indicate wild piHeps, and violet bars indicate CO piHeps. All assays were performed in technical triplicate to ensure reproducibility. Statistical significance was determined by one-way ANOVA followed by Tukey’s post hoc test, and brackets indicate the specific groups compared. * *p* < 0.05; N.S., not significant.

To assess proliferative changes during long-term culture, absolute telomere length and expression of proliferation-associated genes, including *TERT*, *TP53*, *CDKN1A*, and *MYC*, were analyzed across serial passages in EFs and piHeps (Figure 5). At passage 5, telomere length showed no significant difference between EF cells and piHeps. However, distinct divergent trends emerged during subsequent culture. Notably, EFs experienced a statistically significant 1.44-fold sharp decline in telomere length, decreasing from 382 ± 30 at p5 to 265 ± 21 by p21 (*p* < 0.05). In contrast, piHeps effectively maintained or extended their telomeres. By p10, CO piHeps exhibited the most rapid telomere extension, while Wild piHeps showed no significant difference compared to EFs at that stage. From p15 onward, differences in telomere length between piHep types became negligible regardless of the vector type. By p21, the telomere length of CO piHeps (453 ± 25) was significantly 1.71-fold greater than that of age-matched EFs (*p* < 0.05), as vector-related differences among piHeps disappeared (Figure 5A).

Consistent with these changes, *TERT* expression in piHeps increased significantly during culture (*p* < 0.05). Wild piHeps showed a rapid early upregulation at passage 10 (6.2-fold) and reached 14.8-fold at p21 compared to EFs (*p* < 0.05). In contrast, CO piHeps exhibited a more gradual increase, eventually reaching levels comparable to Wild piHeps by p21. Meanwhile, EFs showed a significant 50% decline in TERT expression between p5 and p10, remaining low thereafter (*p* < 0.05, Figure 5B). Expression of cell-cycle regulatory genes also showed concordantly distinct patterns. TP53 exhibited transient changes in piHeps during culture, whereas CDKN1A expression remained relatively low in CO piHeps but significantly increased in EFs over time (*p* < 0.05). *MYC* expression was consistently higher in piHeps compared with EFs across all passages (*p* < 0.05), supporting the enhanced proliferative capacity of piHeps.

## 4. Discussion

In this study, we established expandable hepatocyte-like progenitor cells from GalTKO pigs using a non-integrative reprogramming approach. This system addresses a fundamental limitation of hepatocyte culture, namely restricted proliferative capacity in vitro, by enabling sustained cell expansion. The resulting cells maintained stable growth during extended culture, supporting their use as an expandable and renewable hepatic cell platform.

The use of GalTKO pigs provides a biologically relevant large-animal source with reduced immunogenicity, and the episomal vector system enables reprogramming without genomic integration. By condensing hepatic transcription factors into a minimal two-vector configuration, efficient lineage conversion was achieved while reducing vector complexity. This simplified, non-integrative approach with a reduced vector number enhances biosafety while enabling flexible comparison of vector-dependent effects, providing a safer and more efficient reprogramming strategy compared with conventional viral systems. This strategy enables the evaluation of long-term proliferative behavior, telomere dynamics, and chromosomal stability in a large-animal-derived hepatic cell model. Compared with previous reprogramming studies in human and murine systems, as well as earlier porcine models using lentiviral approaches and alternative factor combinations, this system provides an improved framework for studying proliferative stability and in vitro expansion behavior [10,11,14].

At the transcriptional level, piHeps exhibited activation of hepatic-associated gene expression and suppression of fibroblast-related signatures, indicating a reversal of the mesenchymal state and a transition toward an epithelial-like phenotype. This reprogramming process recapitulated key aspects of hepatic developmental programs and was characterized by a mesenchymal-to-epithelial transition (MET) [9], a critical step in lineage conversion. Induction of key regulators, including HNF4A and GATA6, further supports hepatic lineage commitment [8,16,17]. In addition, increased expression of epithelial markers such as CDH1 and OCLN indicates the establishment of epithelial characteristics associated with hepatic cell identity [16]. Functionally, piHeps exhibited coordinated expression of major xenobiotic metabolism and detoxification pathways, including CYP3A29, the porcine ortholog of human CYP3A4 responsible for metabolizing over half of clinically used drugs [18,19,20], as well as CYP1A2, UGT1A6, and transporters such as ABCA3 and SLC16A1. This relatively stable and broad metabolic gene expression profile distinguishes piHeps from previous porcine hepatic models, which often display variable or incomplete enzyme induction, thereby supporting their applicability in drug metabolism and toxicology studies [10]. Although the induced cells displayed only partial hepatic features, these changes reflect a shift toward hepatic-like identity rather than full maturation.

Chromosomal analysis revealed clear vector-dependent differences in genomic stability, with codon-optimized piHeps exhibiting increased aneuploidy compared with those generated using non-codon-optimized vectors. These findings suggest that enhanced expression efficiency driven by codon optimization may promote cellular metabolic vigor, thereby facilitating the inherent chromosomal plasticity of hepatocytes during long-term culture. While genomic stability is fundamental for reproducibility, this observed variability is consistent with the fact that hepatocyte populations naturally exhibit chromosomal heterogeneity. Hepatic aneuploidy is a pervasive and physiological feature that can promote adaptation to metabolic environments [21,22]. However, the biological significance of the chromosomal alterations observed in piHeps remains to be fully determined.

Supporting this notion, our previous study using a murine system [11] showed even more pronounced chromosomal alterations. In contrast, the relatively stable karyotype observed in the current piHeps shares certain characteristics reported in human hepatocytes; however, direct comparative studies will be required to validate this observation, which maintains a regulated balance of diploid and polyploid populations. This species-specific resemblance highlights the potential value of porcine-derived systems as complementary large-animal models for in vitro studies. Notably, the maintenance of a diploid population alongside these dynamic changes is significant; hepatocytes undergo cycles of polyploidization and ploidy reversal to support liver function, and diploid cells provide the necessary proliferative capacity for effective liver regeneration and repopulation [23].

Consistent with these genomic and species-specific observations, a central finding of this study is the maintenance and modulation of telomere length, accompanied by the coordinated regulation of proliferation-associated genes. Increased expression of TERT and MYC, together with reduced CDKN1A levels, is consistent with enhanced proliferative capacity and an extended cellular lifespan. While TERT expression and telomerase activity have been established as markers of successful reprogramming in porcine induced pluripotent stem cells [24], and the restoration of telomere length has been documented in cloned pigs generated via somatic cell nuclear transfer [25], direct assessment of telomere maintenance in hepatocyte-like cells derived through lineage conversion remains limited. In this context, our findings provide direct evidence that the reprogrammed cells maintain proliferative potential during long-term culture. Importantly, this represents a second and independent line of evidence supporting sustained proliferative capacity, complementing the chromosomal stability and ploidy characteristics described above.

Together, these results highlight the novelty of our system in preserving proliferative capacity in hepatocyte-like cells and underscore its potential as a physiologically relevant and renewable in vitro hepatic model.

## 5. Conclusions

This study presents an expandable hepatocyte-like progenitor cell system derived from a large-animal model. Although full functional maturation remains limited, the ability to maintain proliferation and hepatic-associated characteristics supports the use of these cells as a renewable platform for studying hepatic cell biology, proliferation dynamics, and repeated in vitro applications. Importantly, the sustained proliferative capacity of these cells is supported by multiple independent lines of evidence, including chromosomal stability and telomere maintenance, highlighting the robustness of this system. In addition, the observed species-specific resemblance to human hepatocyte characteristics underscores the value of this porcine-derived model as a physiologically relevant and translationally applicable platform. Future studies should focus on improving genomic stability and defining the biological properties of these cells to optimize their utility as an in vitro platform.

## 6. Patents

A patent related to the generation of proliferative and transdifferentiated hepatocyte-like cells has been granted (Korean Patent No. 10-2010840, October 2019).

## Data Availability

The data supporting the findings of this study are available from the corresponding author upon reasonable request.

## Supplementary Materials

The following supporting information can be downloaded at https://www.mdpi.com/article/10.3390/biology15120958/s1, Figure S1: Hepatic-associated protein expression in piHeps generated with wild or codon-optimized vectors; Figure S2: Validation of α-Gal deletion in porcine genotypes and derived piHeps; Figure S3: Residual vector detection and histochemical characterization of piHeps; Figure S4: Metabolic gene expression profiles in piHeps generated by independent reprogramming batches; Figure S5: Expression of xenobiotic-metabolizing enzymes in long-term cultured piHeps; Table S1: Episomal vectors used for direct hepatic reprogramming of porcine fibroblasts; Table S2: Primer information used in the experiment; Table S3: Details of antibodies used for immunofluorescence staining.
