# Supplementary material for: Telomere-Associated Proliferative Capacity in Expandable Porcine Hepatocyte-like Progenitor Cells
Source: Biology (Basel). 2026 Jun 18;15(12):958. doi: 10.3390/biology15120958 (PMC13296144; doi:10.3390/biology15120958)
Supplement: Supplementary file 1 [file biology-15-00958-s001.zip › Supple tables 260617.pdf]

**Supplementary Table S1. Episomal vectors used for direct hepatic reprogramming of porcine fibroblasts**

| Vector name | Transgene composition | Vector backbone | Codon optimization | Key features | Intended application |
|---|---|---|---|---|---|
| **pEpi-hHNF1A** | Human HNF1A | Episomal (oriP/EBNA1) | Optimized for porcine expression | Non-integrative episomal expression of hepatic transcription factor | Hepatic lineage induction |
| **pEpi-hHNF4A–F2A–hFOXA3** | Human HNF4A linked to human FOXA3 via F2A peptide | Episomal (oriP/EBNA1) | Optimized for porcine expression | Bicistronic expression using self-cleaving F2A peptide | Hepatic lineage induction and maturation |

All episomal vectors were constructed on an oriP/EBNA1 backbone to allow non-integrative and transient gene expression. Coding sequences of human hepatic transcription factors were codon-optimized to enhance expression efficiency in porcine cells. The F2A peptide was used to enable equimolar co-expression of HNF4A and FOXA3 from a single transcript. Full-length coding sequences are available from the corresponding author upon reasonable request.

**Supplementary Table S2. Primer information used in the experiment**

| Gene Name | Type | Sequence | Accession Number |
|---|---|---|---|
| pAFP-1 | F | CGTGCCTCCGCCATTC | NM_214317.1 |
| | R | CTGAGCTTGGCACAGATCCTT | |
| pALB | F | GAAGACACCAGTGAGCGAAAAA | NM_001005208.1 |
| | R | AAGCAAGGCCGTCTGTTCA | |
| pTTR | F | TTCTTACCGTCTGCTCCTCCTT | NM_214212.1 |
| | R | CAGGGCCAGCCTCAGACA | |
| pTF | F | CCGAAGACTGTATCGCCAAGA | X12386.1 |
| | R | TAGCCTCCATCCAAGCTCATG | |
| pTAT | F | CCCCCTCCATTGGCTACCT | XM_003126884.5 |
| | R | CCTCCGGACAGTGGTAATAAGAAG | |
| pVIM | F | AGGAGGCGGAGGAGTGGTA | XM_005668106.2 |
| | R | CATCGTTGTTGCGGTTAGCA | |
| pHNF4A | F | GCTTCTTTCGGAGGAGTGTGA | NM_001044571.1 |
| | R | CACGCACTGCCGACTAAATCT | |
| pHNF1A | F | CAGGGTGGGTTGATTGAAGAG | NM_001032388.1 |
| | R | CGCCCCTTCTTGGTTGGT | |
| pFOXA3 | F | ATGCACCCTACAACTTCAACCA | XM_003127229.3 |
| | R | TGGCGTCTGCTCCGACAT | |
| hHNF1A | F | CGGAGGAACCGTTTCAAGTG | NM_000545.6 |
| | R | AGGGTTCTTCTGCCTCTCATAGG | |
| hHNF4A | F | CGCTACTGCAGGCTCAAGAAA | NM_000457.4 |
| | R | TCTGGACGGCTTCCTTCTTC | |
| hFOXA3 | F | AGCAGCGCTGGCAGAACT | NM_004497.2 |
| | R | CACCTTGACGAAGCAGTCGTT | |
| hHNF1A **porcine opti** | F | TGGGAGAGACACGCGGCAG | NM_000545.6 |
| | R | GGGGTGAAGTCCTCGCCG | |
| hHNF4a **porcine opti** | F | AGATCAAGCGCCTGCGCAG | NM_000457.4 |
| | R | AAGCGGCCCCTGCTGTCG | |
| hFOXA3 **porcine opti** | F | CCTGAACCCCCTGAGCAGC | NM_004497.2 |
| | R | GCAGCTGGAGCTGGTGGG | |
| F2A | F | AGCCCCCGTGAAGCAGAC | |
| | R | CCTGGGTTGCTTTCCACGTC | |
| pCYP3A29 | F | AGGAGGAGATTGAGGCAACTTTC | NM_214423.1 |
| | R | TCTGTGCCAGGGCATCGTA | |
| pCYP2E1 | F | TTACGGGCCGGTGTTCACT | NM_214421 |
| | R | TAGCCGTGCAGGACCACAA | |
| pCYP1A2 | F | GGCAGGGCGACGATTTC | NM_001159614.1 |
| | R | TCAGTGACCAGAGTGAAGCTGTAGA | |
| pCDKN1A | F | CCTCCCAGGGCAGGAAA | XM_013977858.2 |
| | R | GCGTTTGGAGTGGTAGAAATCTG | |
| pTERT | F | CAC TGC GAC TAT GCC AGT TAC G | AY785158.1 |
| | R | GCC CTG GTT GAA GGT GAG ACT | |
| pTP53 | F | TCC GGG TGG AAG GGA ATT | NM_213824.3 |
| | R | ACA ACG CTG TGT CGA AAA GTG T | |
| pMYC | F | TCTGCCAAGAGGGCTAAGTTG | NM_001005154.1 |
| | R | CGGTTGTTGCTGATCTGTTTCA | |
| pHPRT1 | F | CCA TCT TCC AGG AGC GAG ATC | NM_001206359.1 |
| | R | GCC TTC TCC ATG GTC GTG AA | |

**Supplementary Table S3. Details of antibodies used for immunofluorescence staining.**

| Proteins | First antibody | | Secondary antibody | |
|---|---|---|---|---|
| | Information on antibodies | Dilution factor | Information on antibodies | Dilution factor |
| CYP1A1 | CYP1A1 Antibody (1A3-03) sc-101828 (Santa Cruz Biotechnology) | 1:100 | m-IgGκ BP-FITC sc-516140 (Santa Cruz Biotechnology) | 1:100 |
| CYP2A | CYP2A Antibody (C-20) sc-9896 (Santa Cruz Biotechnology) | 1:100 | Donkey anti-goat IgG-FITC sc-2024 (Santa Cruz Biotechnology) | 1:200 |
| CYP3A4 | Rabbit Cytochrome P450 Enzyme CYP3A4 Polyclonal Antibody MBS615441 (MyBioSource) | 1:200 | Goat anti-rabbit IgG-FITC sc-2012 (Santa Cruz Biotechnology) | 1:200 |
| Albumin | Anti-Albumin antibody (ab112980) (Abcam) | 1:500 | Donkey anti-goat IgG-FITC sc-2024 (Santa Cruz Biotechnology) | 1:200 |
| AAT | Alpha-1 Antitrypsin Polyclonal Antibody PA1-22860 (Thermo Fisher Scientific) | 1:500 | Goat anti-rabbit IgG-FITC sc-2012 (Santa Cruz Biotechnology) | 1:200 |
| Transferrin | Transferrin Monoclonal Antibody (HTF-14) MA1-19013 (Thermo Fisher Scientific) | 100X | Goat anti-Mouse IgG (H+L) Highly Cross-Adsorbed Secondary Antibody, Alexa Fluor Plus 555 A32727 (Thermo Fisher Scientific) | 1:1000 |
| E-cadherin | E-cadherin (N-20) sc-1500 (Santa Cruz Biotechnology) | 1:200 | Donkey anti-goat IgG-FITC sc-2024 (Santa Cruz Biotechnology) | 1:200 |
