# Supplementary material for: Telomere-Associated Proliferative Capacity in Expandable Porcine Hepatocyte-like Progenitor Cells
Source: Biology (Basel). 2026 Jun 18;15(12):958. doi: 10.3390/biology15120958 (PMC13296144; doi:10.3390/biology15120958)
Supplement: Supplementary file 1 [file biology-15-00958-s001.zip › Supplementary figure legends.pdf]

Supplementary figure legends

Supplementary Figure S1. Hepatic-associated protein expression in piHeps generated with wild or codon-optimized vectors.

(A) Confocal immunofluorescence images of wild piHeps generated using non–codon-optimized vectors. Left panels show cytochrome P450 enzymes (*CYP1A1, CYP2A, CYP3A1*, and *CYP3A29*), and right panels show hepatocyte-associated proteins (albumin, alpha-1-antitrypsin, transferrin, and E-cadherin). (B) Confocal immunofluorescence images of codon-optimized (CO) piHeps showing the same markers. Nuclei were counterstained with DAPI. Scale bar, 50 μm.

Supplementary Figure S2. **Validation of α-Gal deletion in porcine genotypes and derived piHeps**

Genomic PCR analysis confirming the GGTA1 (α-Gal) genotype of donor pigs and co rresponding piHeps. Homozygous GGTA1 knockout samples (GalT^{-}/$^{-}$) show a single 3 .6 kb band, heterozygous samples (GalT^{+}/$^{-}$) show 3.6 kb and 2.3 kb bands, and wild samples (GalT^{+}/$^{+}$) show a single 2.3 kb band. piHeps retained genotypes consistent wi th their donor fibroblasts.

Supplementary Figure S3. Residual vector detection and histochemical characterization of piHeps

(A) Relative expression of F2A three months after differentiation, indicating residual episomal vector presence. Ear fibroblasts (EF) served as negative controls. (B–E) Histochemical and cellular assays performed on piHeps differentiated for four weeks at passage 2: (B) PAS staining for glycogen storage, (C) Dil-ac-LDL uptake, (D) indocyanine green (ICG) uptake (D-1) and release (D-2), and (E) Oil Red O staining for lipid accumulation. Upper panels show

wild piHeps and lower panels show CO piHeps. (F) Urea production measured using a commercial assay kit. Data are presented as mean ± SEM from triplicate experiments. Scale bar, 100 μm. *$p < 0.05$; N.S., not significant.

Supplementary Figure S4. Metabolic gene expression profiles in piHeps generated by independent reprogramming batches

Microarray-based gene expression analysis of metabolic pathways in porcine induced piHeps generated using wild or codon-optimized (CO) episomal vectors.

(A) Fatty acid metabolism–related gene expression profiles. (B) Glucose and glucagon metabolism–related gene expression profiles. (C) Heatmap of lipoprotein- and cholesterol-associated gene expression. A two-fold cutoff was applied to define differential gene expression. Sample groups are color-coded to represent cell type, vector type, maturation stage, and independent reprogramming batches derived from the same donor pig.

Supplementary Figure S5. Expression of xenobiotic-metabolizing enzymes in long-term cultured piHeps.

Relative expression of (A*) CYP1A2* and (B) *CYP3A29* in piHeps cultured for three months. Ear fibroblasts (EF) served as negative controls. Data are presented as relative quantification (RQ) values with minimum and maximum ranges. Experiments were performed in triplicate. *$p < 0.05$.
