# Supplementary figures and images for: Telomere-Associated Proliferative Capacity in Expandable Porcine Hepatocyte-like Progenitor Cells
Source: Biology (Basel). 2026 Jun 18;15(12):958. doi: 10.3390/biology15120958 (PMC13296144; doi:10.3390/biology15120958)

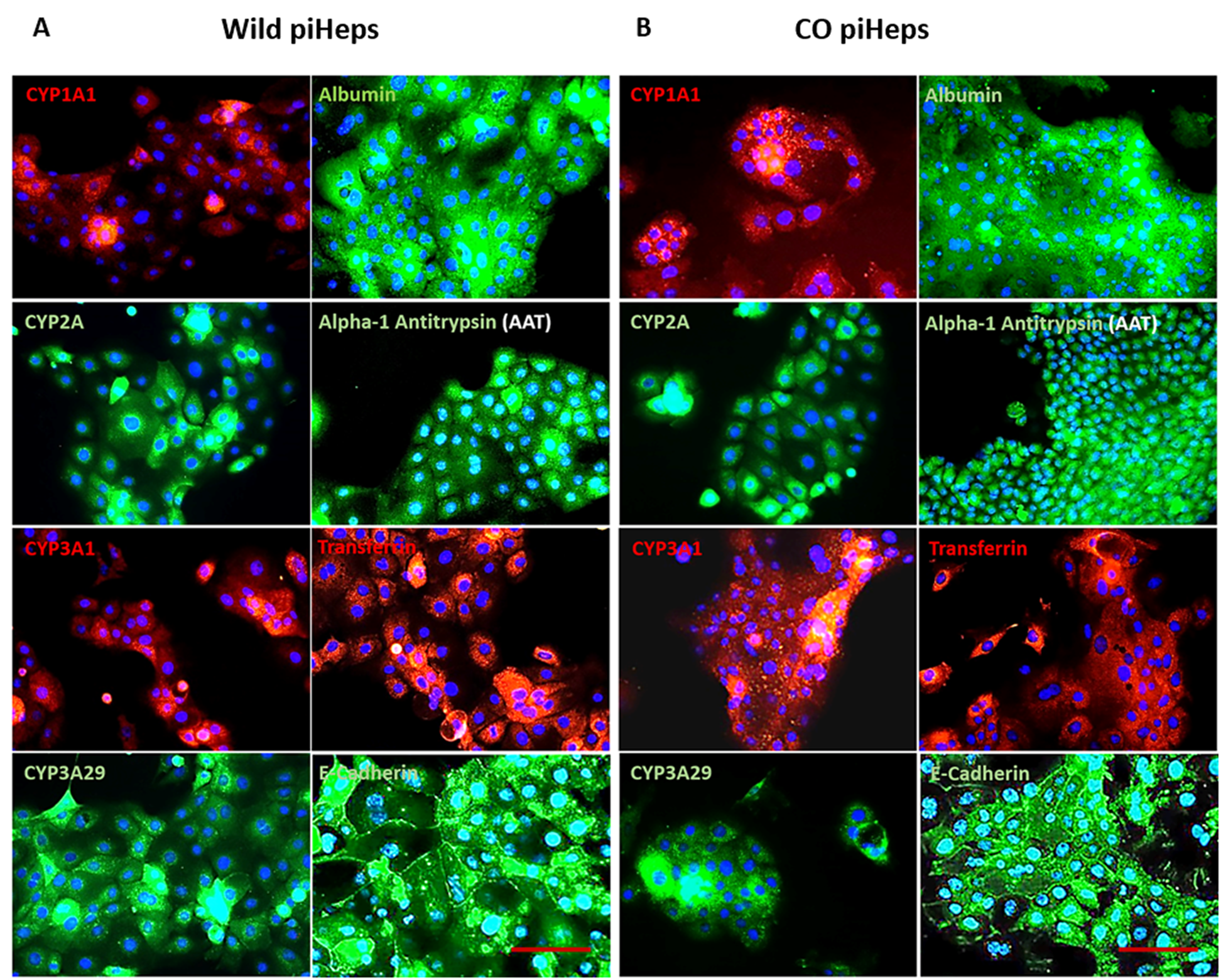

Supplement: Supplementary file 1 [file biology-15-00958-s001.zip › Figure S/FIG S1.tif]

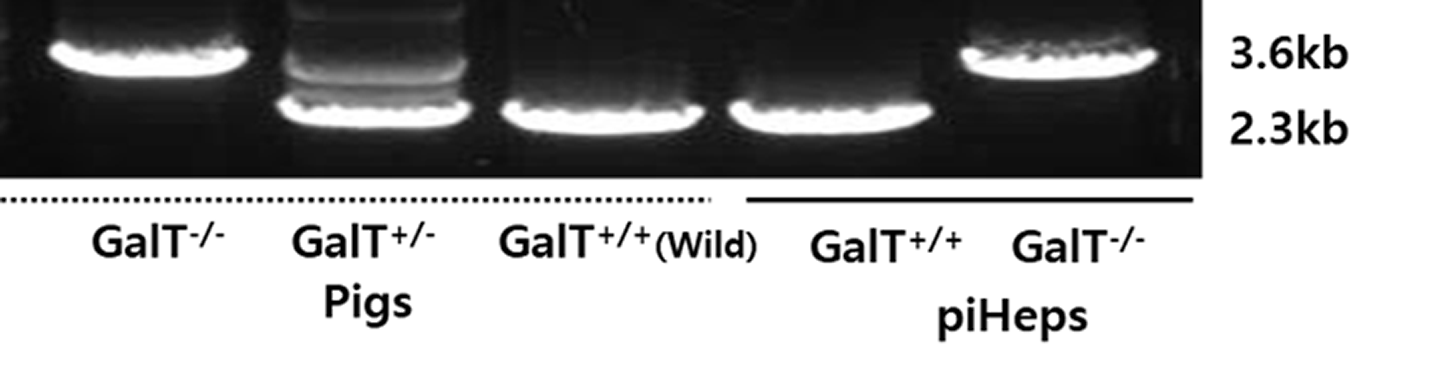

Supplement: Supplementary file 1 [file biology-15-00958-s001.zip › Figure S/FIG S2.tif]

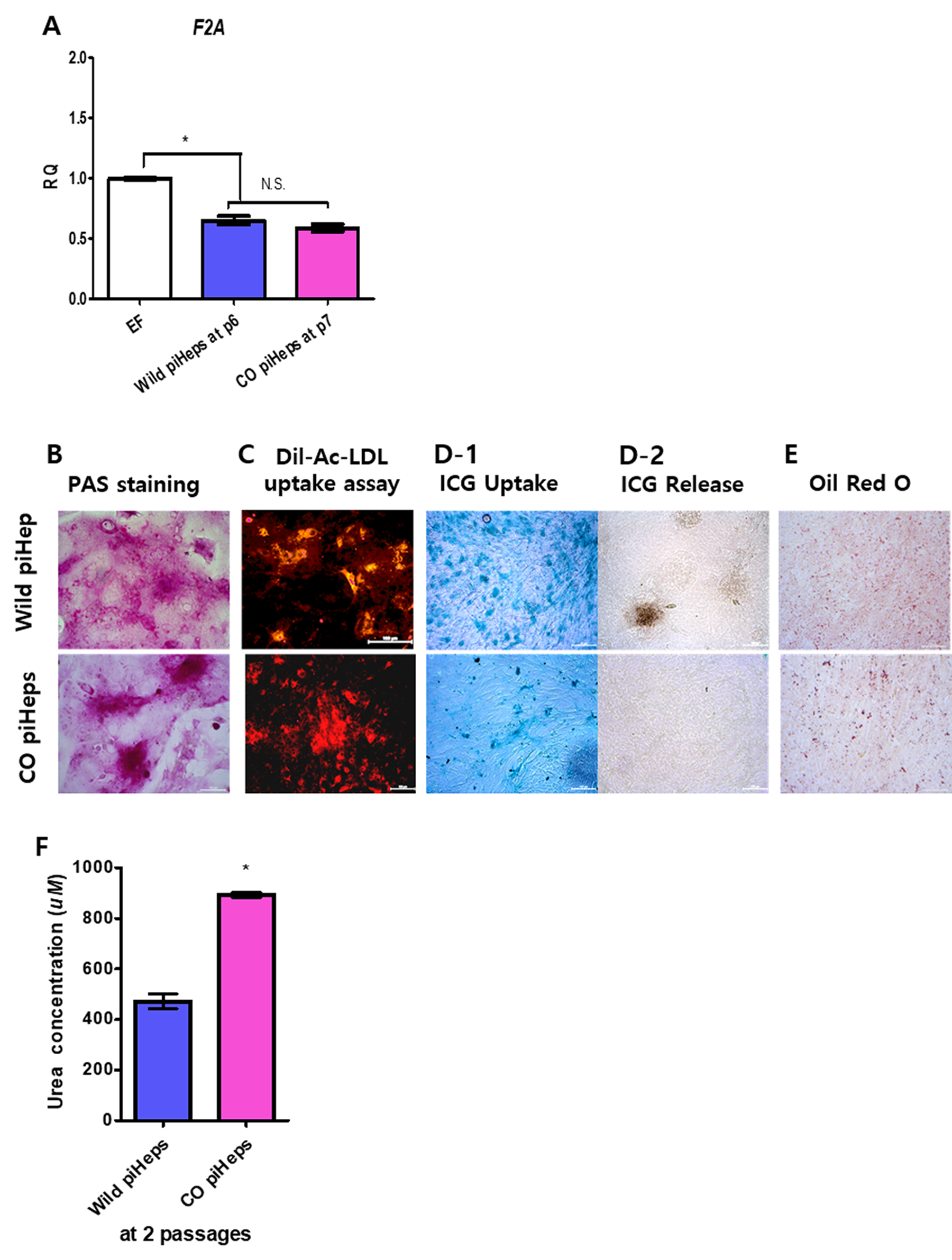

Supplement: Supplementary file 1 [file biology-15-00958-s001.zip › Figure S/FIG S3.tif]

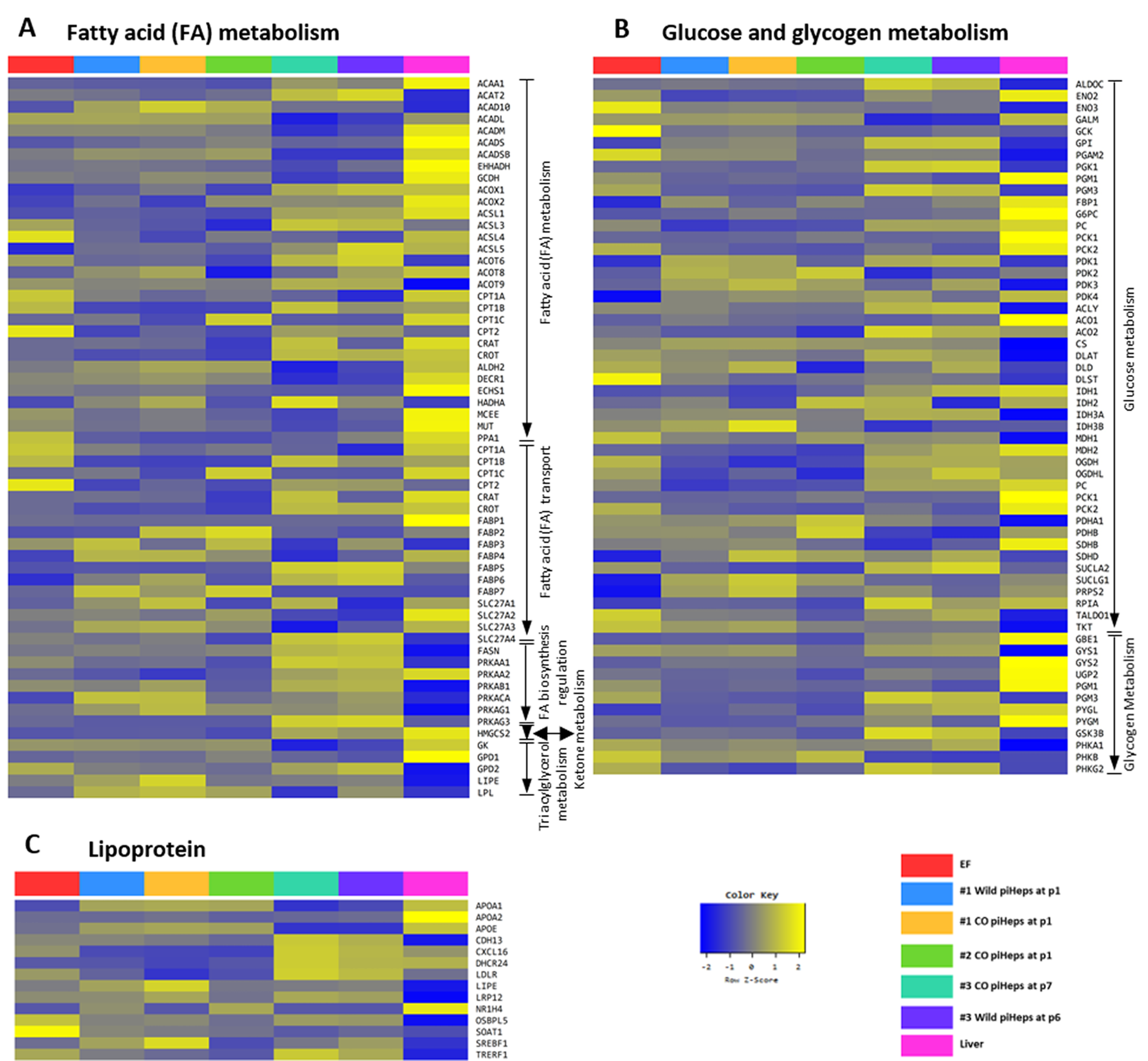

Supplement: Supplementary file 1 [file biology-15-00958-s001.zip › Figure S/FIG S4.tif]

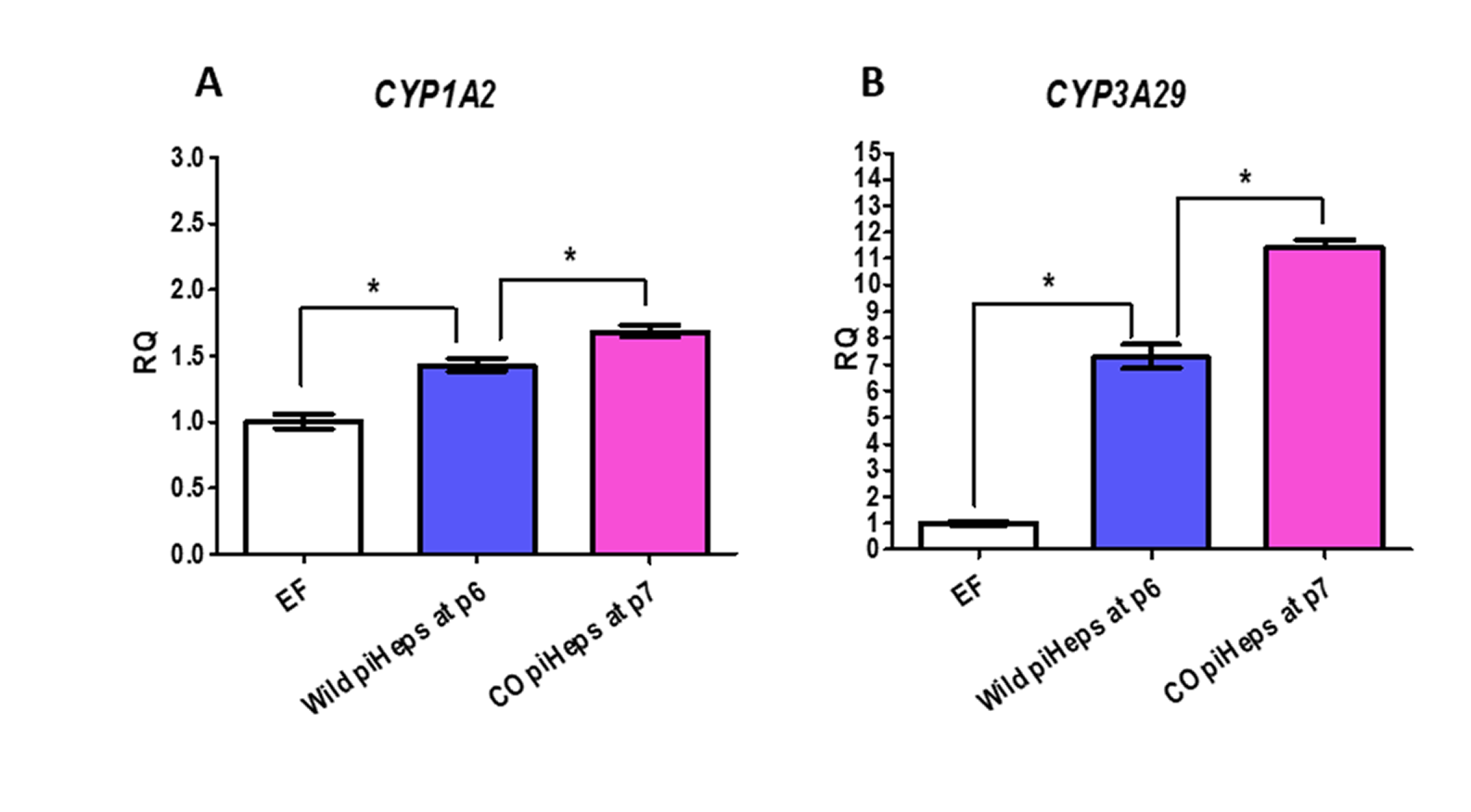

Supplement: Supplementary file 1 [file biology-15-00958-s001.zip › Figure S/FIG S5.tif]
